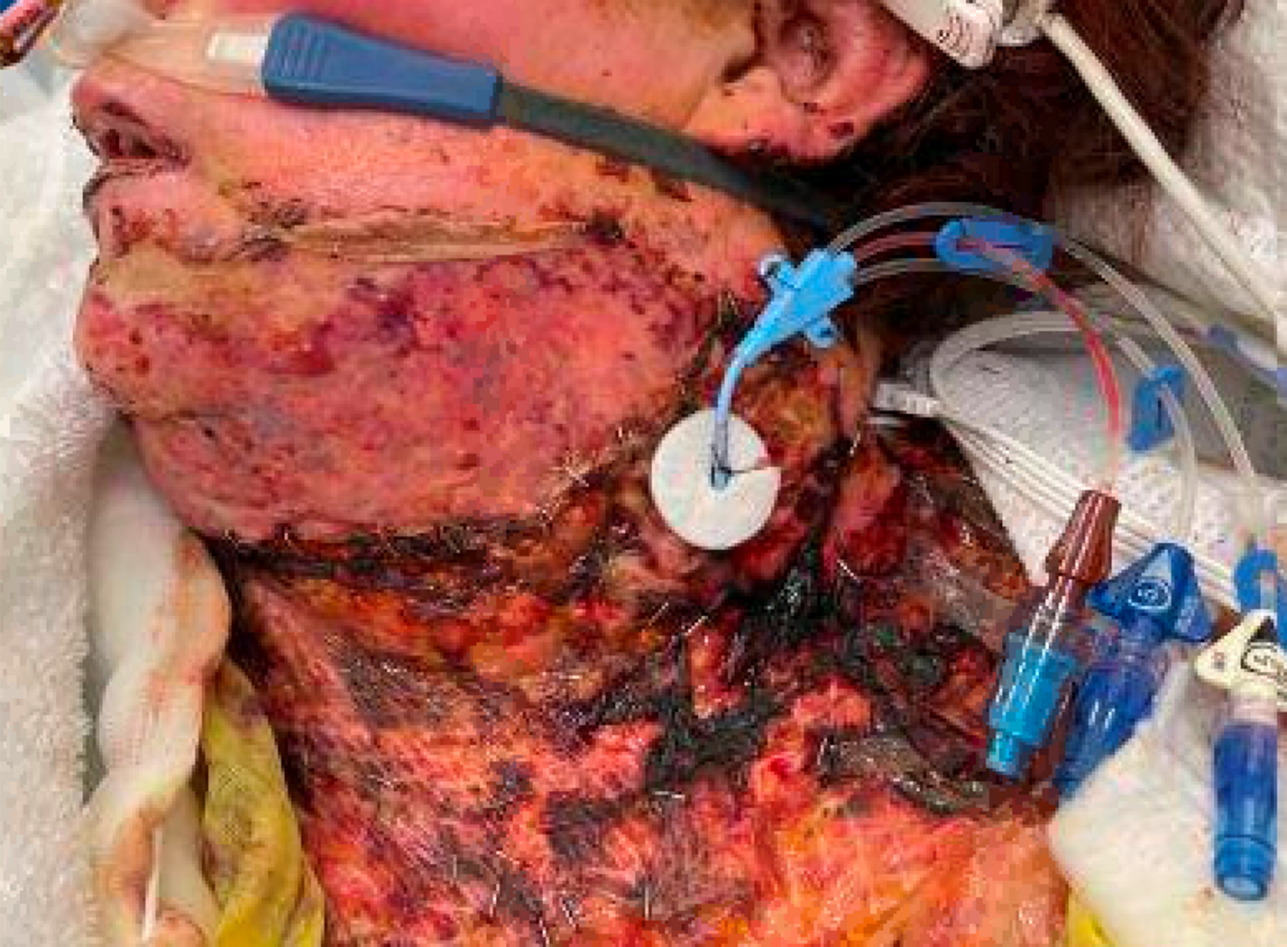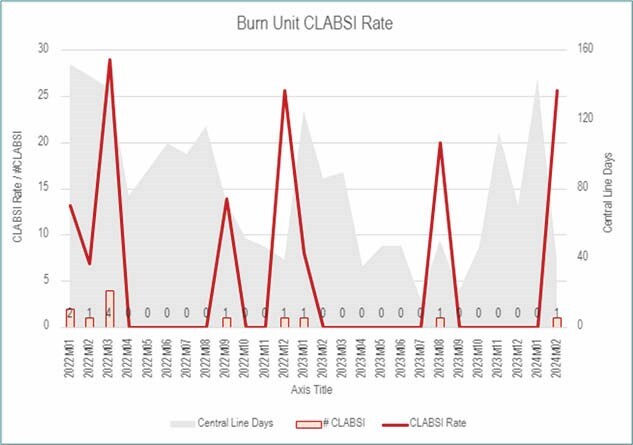# 986 The Reduction of CLABSI’s with the Use of Silver-Plated Disk Dressings in Burn Injured Patients

**DOI:** 10.1093/jbcr/iraf019.517

**Published:** 2025-04-01

**Authors:** Michaela Craig, Sendra Starnes, Tamika Lovelace, James Holmes

**Affiliations:** Wake Forest Baptist Medical Center Burn Center; Wake Forest Baptist Medical Center Burn Center; Wake Forest Baptist Medical Center Burn Center; Wake Forest University

## Abstract

**Introduction:**

Burn patients are particularly more susceptible to infections due to compromised skin integrity, frequent exposure to moisture during wound care, and elevated ambient temperatures in care settings, which are maintained to mitigate the risk of hypothermia. Additionally, there is an elevated prevalence of central-line associated bloodstream infections, (CLABSIs) among burn patients. This increased CLABSI risk/prevalence underscores the critical interplay between these vulnerabilities and the heightened risk of infection. This correlation highlights the need for targeted interventions to reduce the incidence of CLABSIs in this particularly at-risk population. This study investigates the efficacy of silver-plated disk dressings in reducing CLABSIs within a single burn center.

**Methods:**

To address the high risk, and relatively increased prevalence of CLABSIs, a simple-intervention QI/PI process was implemented within the Burn Intensive Care Unit (ICU) of a single ABA-verified Burn Center, beginning in June 2022. The initiative included the adoption of a silver-plated disk as the primary dressing for all arterial and central venous access devices. Dressing changes were scheduled biweekly. The effectiveness of this intervention was evaluated by assessing the rates of CLABSIs before and after its implementation.

**Results:**

From January 2022 to June 2022, a total of 7 CLABSIs were recorded over 808 line-days (event rate: 0.0087). Following the implementation of the silver-plated disk dressing, a significant reduction of CLABSIs was observed from June 2022 to February 2024, 5 CLABSIs over 1,264 line-days (event rate: 0.0040). A sixth CLABSI was observed but excluded from these analyses due to nursing noncompliance with dressing protocols, thereby classifying it as an outlier. A calculated z-score of 1.37 indicates a significant impact on CLABSI reduction (p< 0.05) following the implementation of silver disk technology.

**Conclusions:**

The adoption of silver-plated disk dressings has effectively decreased CLABSIs in adult burn patients within the ICU setting, as compared to the standard of care CHG dressings previously used. The kits, initially crafted by hand and assembled in-house, have evolved significantly. They are now professionally packaged and distributed by a regional distributor, thus allowing for standardization of their central line dressing kit to include the silver-plated disk and marking a significant advancement in patient care practices within the Burn ICU. Ongoing institutional policy revisions reflect this practice change, emphasizing the dressing’s role in setting new standards of care.

**Applicability of Research to Practice:**

The silver-plated disk dressing has demonstrated a benefit in reducing CLABSIs among adult burn patients, as compared to the standard of care CHG dressings previously used. This reduction in infections is likely due to silver’s antimicrobial properties and better site adherence under challenging conditions.

**Funding for the Study:**

N/A